# Microvolt T wave alternans in adults with congenital heart diseases characterized by right ventricle pathology or single ventricle physiology: a case control study

**DOI:** 10.1186/1471-2261-13-26

**Published:** 2013-04-03

**Authors:** Aleksandra Cieplucha, Olga Trojnarska, Agnieszka Bartczak, Lucyna Kramer, Stefan Grajek

**Affiliations:** 1 1st Department of Cardiology, University of Medical Sciences, Poznan, Poland; 2 Department of Computer Sciences and Statistics, University of Medical Sciences, Poznan, Poland

## Abstract

**Background:**

Among adults with congenital heart diseases (CHD) evaluation of sudden cardiac death (SCD) risk remains a great challenge. Although microvolt T-wave alternans has been incorporated into SCD risk stratification algorithm, its role in adults with CHD still requires investigation. We sought to determine the incidence of MTWA in this specific group and its coincidence with ventricular arrhythmia (VA) and other clinical findings presumably associated with SCD.

**Methods:**

A case–control study was performed in which 102 patients with CHD characterized by right ventricle pathology or single ventricle physiology (TGA, UVH, Ebstein’s anomaly, ccTGA, Eisenmenger syndrome, DORV, CAT, unoperated ToF) were compared to 45 age- and sex-matched controls. All subjects underwent spectral MTWA test, ambulatory ecg monitoring, cardiopulmonary test, BNP assessment. After excluding technically inadequate traces, the remaining MTWA results were classified as positive(+), negative(−) and indeterminate(ind). Due to similar prognostic significance MTWA(+) and (ind) were combined into a common group labeled ‘abnormal’.

**Results:**

Abnormal MTWA was present more often in the study group, compared to controls (39.2% vs 2.3%, p = 0.00001). Sustained ventricular tachycardia (sVT) was observed more often among subjects with abnormal MTWA compared to MTWA(−): 19.4% vs 3.6%, p = 0.026. The patients with abnormal MTWA had a lower blood saturation (p = 0.047), more often were males (p = 0.031), had higher NYHA class (p = 0.04), worse cardiopulmonary parameters: %PeakVO_2_ (p = 0.034), %HRmax (p = 0.003). Factors proven to increase probability of abnormal MTWA on multivariate linear regression analysis were: sVT (OR = 20.7, p = 0.037) and male gender (OR = 15.9, p = 0.001); on univariate analysis: male gender (OR = 2.7, p = 0.021), presence of VA (OR = 2.6, p = 0.049), NYHA > I (OR = 2.06, p = 0.033), %HRmax (OR = 0.94, p = 0.005), %PeakVO_2_ (OR = 0.97, p = 0.042), VE/VCO_2_slope (OR = 1.05, p = 0.037).

**Conclusions:**

Abnormal MTWA occurs significantly more often in adults with the chosen forms of CHD than among healthy subjects. The probability of abnormal MTWA increases in patients with malignant VA, in males and among subjects with heart failure and cyanosis. MTWA might be of potential role in risk stratification for SCD in adults with CHD.

## Background

A dynamic progress of pediatric cardiosurgery made survival to adulthood possible for most patients with congenital heart defects (CHD) [1-4]. Most die due to cardiovascular complications including a sudden cardiac death (SCD) that accounts for 19%-30% of total mortality and is observed mainly in patients with complex forms of CHD [1-6]. Majority, i.e. 75%-80%, of SCD is caused by ventricular fibrillation [5,6] but still this heterogenous population lacks fine indications for implantable cardioverter-defibrillator (ICD) therapy in primary prophylaxis [7]. Ventricular arrhythmia (VA) among these patients originates from myocardial fibrosis due to cyanosis and insufficient intraoperative cardioprotection; scars after ventriculotomy; hemodynamic changes resulting in ventricular dilation and increased wall stress, consequentially causing areas of electrical inhomogeneity within myocardium [2,8]. Noninvasive risk stratification for arrhythmic SCD is based on factors that possibly contribute to the aforementioned pathological chain that includes presence of malignant VA during ecg ambulatory monitoring [5,9], heart failure [10-12], or widening of QRS complex reflecting intra- and interventricular delay [9]. Nevertheless, such management still lacks accuracy.

Microvolt T wave alternans (MTWA) – an electrocardiographic phenomenon displaying inhomogeneity of the myocardial repolarization process is a promising method of VA risk evaluation in patients with ischemic and non-ischemic cardiomyopathies [13-15], which has been recently included into guidelines for the SCD prevention (class IIa) [6]. It has been proven that the heterogeneity of the intracardiac repolarization leads to dispersion of the depolarizing wave, reentry phenomenon and, as a consequence, initiation of the ventricular arrhythmia [6]. To our best knowledge, there have been merely two studies on spectral MTWA phenomenon in CHD patients published so far, both concerning only pediatric population [16,17]. Therefore, the aim of our study was to determine the incidence of spectral MTWA among grown-up patients with the chosen forms of CHD characterized by pathology within right ventricle or single ventricle physiology. Moreover, we evaluated the coincidence between MTWA and malignant and potentially malignant ventricular arrhythmia, as well as other demographic and clinical findings presumably associated with an increased risk of malignant VA and SCD in the analyzed population.

## Methods

The study group consisted of 102 consecutive patients (47 males) in sinus rhythm diagnosed with CHD characterized by pathology within right ventricle or single ventricle physiology, aged 18-75 years (mean 34.2 ± 13.6 years), of whom 46 (45.1%) were operated on at the age of 0.5-32 years (mean 6.2 ± 6.3 years). None of them had indications for ICD in secondary prophylaxis. Five patients (4.9%) presented with common arterial trunk (CAT), 9(8.8%) – congenitally corrected transposition of the great arteries (ccTGA), 6(5.9%) – double outlet right ventricle (DORV), 32(31.4%) - Ebstein’s anomaly, 9(8.8%) – Eisenmenger syndrome due to simple shunt lesions, 20(19.6%) – complete transposition of the great arteries (TGA), 3(2.9%) – native tetralogy of Fallot (ToF), 18(17.7%) – univentricular heart (UVH) including 8 patients with tricuspid atresia, 3 – ccTGA, 4 – double inlet left ventricle (DILV) and 3 – common atrioventricular canal (CAVC). Surgical correction was performed in 2 (40%) patients with CAT, 6 (100%) with DORV, 2 (6.2%) with Ebstein anomaly, 18 (90%) with TGA (using method of intraatrial repair), 12 (66.6%) with UVH (including 11 Fontan operations and 1 pulmonary banding). The control group consisted of 45 healthy volunteers (23 males) aged 18-65 years (mean 31.0 ± 9.2 years). The exclusion criteria were: persistent atrial fibrillation, permanent cardiac pacing, serum creatinine > 140 μmol/l, serum aspartate transaminase level exceeding twice the upper limit of normal, pulmonary diseases, mental or physical disability preventing cooperation. Functional status was assessed according to the New York Heart Association (NYHA) classification. Pulse oximetry was used to measure resting blood oxygen saturation (SO_2_).

Microvolt T wave alternans was analyzed in 102P during treadmill test while receiving their usual medications, including beta blocker therapy [18]. After careful skin preparation high-resolution electrodes (Micro-V Alternans Sensors, Cambridge Heart Inc.) were applied using Frank orthogonal configuration. MTWA was recorded during a heart rate (HR)-based exercise protocol using CH2000 (Cambridge Heart Inc., Bedford, MA, USA) with the use of spectral method [14]. The test was considered positive if the significant MTWA (i.e. alternans voltage ≥ 1.8 μV and alternans ratio > 3.0) appeared at an onset HR ≤ 110 bpm, was present for ≥1 min in any orthogonal lead or two adjacent precordial leads and sustained as long as HR remained above the patient specific onset HR. The test was classified as negative if sustained MTWA was absent at HR ≤ 110 bpm and if there was ≥1 min at HR ≥ 105 bpm of artifact-free trace (noise level < 1.8 μV, ectopy < 10%). Otherwise, the test was considered indeterminate and immediately repeated in order to reduce the number of such results. As generally accepted [18] indeterminate test may be caused either by patient factors (including: 1) excessive ectopy during exercise, 2) unsustained MTWA, 3) failure to achieve HR = 105 bpm) or technical factors (including: 1) a noisy recording, 2) failure to maintain HR between 105-110 bpm for ≥1 min). The indeterminate results due to technical factors were ultimately excluded from further analysis. Considering similar prognostic significance of tests classified as positive or indeterminate due to patient factors they were combined into a common group labeled ‘non-negative’ [MTWA(non-)] i.e. abnormal.

Transthoracic echocardiography was performed with VIVID 7 GE Medical System device, using 1.5-2.5Hz probe in 2D, M and Doppler modes. The diagnoses of anatomical anomalies were set according to the current recommendations of the European Society of Cardiology (ESC) [7]. Alike in other studies [19] the diagnosis of pulmonary arterial hypertension (PAH) was based on echocardiography. The estimation of pulmonary arterial pressure (PAP) was based on the peak velocity of the jet of tricuspid regurgitation enabling to calculate right ventricular systolic pressure (using the simplified Bernoulli equation). After excluding pulmonary stenosis, PAP was calculated as the sum of right ventricular systolic pressure and right atrial pressure (estimated as 5-15 mmHg depending on the diameter and respiratory variation of the inferior vena cava). According to the current guidelines on PAH management, we considered the criterion of estimated PAP ≥ 37 mmHg combined with additional echocardiographic variables suggestive of PAH a sufficient basis to set this diagnosis [20].

Cardiopulmonary exercise treadmill test was performed according to the modified Bruce protocol (adding stage 0-3 min, 1.7 km/h, 5% grading) and symptom limited. The equipment was calibrated with a standard gas mixture before each test. Patients were encouraged to continue the exercise until the respiratory exchange ratio (RER) exceeded 1. Oxygen uptake (PeakVO_2_), carbon dioxide production (VCO_2_) and minute ventilation were measured with breath by breath technique using Sensor Medics, model V_max_29. Peak VO_2_ was defined as the average for the last 20s of exercise and expressed as ml/kg/min and ml/min, as well as the percentage ranges predicted for sex, age, height and weight [21]. A ventilation/carbon dioxide slope (VE/VCO_2_ slope) was assessed by linear regression for the whole exercise. A standard 12-lead electrocardiography was continuously recorded. Due to physical disability cardiopulmonary test was not performed in three patients.

Ambulatory 24-hour ECG monitoring was registered the same day as other tests using system CardioScan12.0 Premier, Oxford, 12.4.0040a version. Additionally, all previous results of ambulatory ECG available in the patient’s medical records were analyzed for presence of VA, which was classified as: excessive ventricular ectopy (ventricular premature beats – VPB ≥ 10/hour), episodes of nonsustained ventricular tachycardia – nsVT (presence of ≥3 consecutive ventricular beats at a rate greater than 100 bpm, terminating spontaneously in less than 30s), episodes of sustained ventricular tachycardia – sVT (ventricular tachycardia greater than 30s in duration or hemodynamically unstable) [6]. According to the classification regarding patients with structural heart disease [6,22] this findings were divided into two groups: potentially malignant ventricular arrhythmia (VPB ≥ 10/hour and/or nsVT) and malignant ventricular arrhythmia (sVT). For the purposes of analysis, a group comprising of patients with any of the specified forms of VA was created and labeled ‘VPB ≥ 10/hour/nsVT/sVT’.

QRS duration was adopted as the longest value measured among all 12 leads in standard electrocardiogram.

Serum brain natriuretic peptide (BNP) level was measured in the venous blood samples taken after 15 min of rest in supine position using the Abbott AxSYM Immunoassay system.

Statistical analysis. Continuous variables were summarized by mean ± SD or median and minimal-maximal values, depending on normality of distribution assessed with Wilk-Shapiro test. Variables following normal distribution were analyzed using Student’s *t* test or Welch test, otherwise nonparametrical U Mann–Whitney test comparing two groups was used. Categorical variables are represented by frequencies and percentages. For 2 × 2 tables chi-square test with Yates’s correction or Fisher’s exact test were performed. Factors associated with non-negative MTWA were assessed by logistic regression. Because some subgroups were too small it was impossible to perform multivariable logistic regression model for all parameters entered into analysis. Two-tailed values of p < 0.05 were considered statistically significant. Analyses were performed using the Statistica 7.0 package.

Informed consent was obtained from all subjects. Our study protocol, as approved by the Ethics Committee of University of Medical Sciences in Poznan, conformed to the ethical guidelines of the 1975 Declaration of Helsinki.

## Results

The MTWA examination was performed in total of 102 patients and 45 healthy individuals. The MTWA(+) as well as MTWA(ind) was significantly more frequent in the study group than among controls (respectively: 18(17.6%) vs 1(2.2%), p = 0.008 and 18(17.6%) vs 0(0.0%), p = 0.002) (Table 1). Ultimately, 10 patients and 2 controls with MTWA indeterminate due to technical factors were excluded from further calculations and the rest constituted two groups under analysis.

**Table 1:** Microvolt T wave alternans in the study and control groups

	**Study group (n = 102)**	**Control group (n = 45)**	**Study group vs control group p**
**MTWA(+) (%)**	18 (17.6)	1 (2.2)	**0.008**
**MTWA(ind) (%)**	18 (17.6)	0 (0)	**0.002**
**MTWA(noise) (%)**	10 (9.8)	2 (4.4)	0.35
**MTWA(−) (%)**	56 (54.9)	42 (93.4)	**0.00001**
**MTWA(non -) (%)**	36 (35.3)	1 (2.2)	**0.00001**

MTWA(+) – positive result; MTWA(ind) - result indeterminate due to patient factors; MTWA(noise) – result indeterminate due to technical factors; MTWA(−) –negative result; MTWA(non-) – non-negative (abnormal) result.

As shown in Table 2 the number of males among patients with MTWA(+) and MTWA(non-) was significantly higher than in MTWA(−) group (respectively: 72.2% vs 33.9%, p = 0.006 and 58.3% vs 33.9%, p = 0.03). Patients with MTWA(non-) presented a higher NYHA class comparing to those with MTWA(−) [2.0(min1.0-max3.0) vs 1.0(min1.0-max3.0), p = 0.04] and lower resting SO_2_ [94.5%(min73-max100) vs (97%(min76-max100), p = 0.047].

**Table 2:** Relationship between MTWA result and demographic or clinical features in the analyzed group

	**Analyzed group (n=92)**	**MTWA (+) (n = 18)**	**MTWA (ind) (n = 18)**	**MTWA (−) (n = 56)**	**MTWA (non-) (n = 36)**	**MTWA(+) vs MTWA(−) p**	**MTWA(non-) vs MTWA(−) p**
**Males (%)**	40 (43.5)	13 (72.2)	8 (44.4)	19 (33.9)	21 (58.3)	0.006	0.031
**Age (years)**	29	28	31,5	29	29.5	0.782	0.620
Median	(18–75)	(19–64)	(20–75)	(18–74)	(19–22)		
Min-max							
**Operated (%)**	28 (27.5)	9 (50)	4 (22.2)	9 (50)	13 (36.1)	0.786	0.663
**NYHA**	1.0	2.0	2.0	1.0	2.0	0.231	0.040
Median	(1.0-3.0)	(1.0-3.0)	(1.0-3.0)	(1.0-3.0)	(1.0-3.0)		
Min-max							
**SO**_**2 **_**(%)**	97	96	92.5	97	94.5	0.151	0.047
Median	(69–100)	(75–99)	(73–99)	(69–100)	(73–100)		
Min-max							
**Pulmonary hypertension (%)**	11 (12.0)	1 (5.6)	7 (38.9)	9 (16.1)	2 (5.6)	0.434	0.191
**Beta blocker (%)**	25 (27.1)	7 (38.9)	3 (16.7)	15 (26.8)	10 (27.8)	0.380	1.0
**QRS (ms)**	120	120	135	120	125	0.354	0.123
Median Min-max	(80–200)	(100–200)	(100–180)	(80–200)	(100–200)		

MTWA(+) – positive result; MTWA(ind) – indeterminate result; MTWA(−) –negative result; MTWA(non-) – non-negative result; SO_2_ – blood oxygen saturation.

Malignant ventricular arrhythmia occured significantly more often in patients with abnormal compared to negative MTWA [7(19.4%) vs (2(3.6%), p = 0.026]. However, comparison between incidence of other forms of VA among subgroups characterized by different MTWA result did not show statistical significance (Table 3).

**Table 3:** Relationship between MTWA result and incidence of ventricular arrhythmia in the analyzed group

	**Analyzed group (n = 92)**	**MTWA (+) (n = 18)**	**MTWA (ind) (n = 18)**	**MTWA (−) (n = 56)**	**MTWA (non-) (n = 36)**	**MTWA(+) vs MTWA(−) p**	**MTWA(non-) vs MTWA(−) p**
**VPB ≥ 10/h/nsVT (%)**	23 (25.0)	3 (16.7)	9 (50.0)	11 (19.6)	12 (33.3)	1.0	0.217
**sVT (%)**	9 (9.9)	1 (5.7)	6 (33.3)	2 (3.6)	7 (19.4)	1.0	**0.026**
**VPB ≥ 10/h/nsVT/sVT (%)**	27 (29.3)	4 (22.2)	11 (61.1)	12 (21.8)	15 (41.7)	1.0	0.060

MTWA(+) – positive result; MTWA(ind) – indeterminate result; MTWA(−) – negative result; MTWA(non-) – non-negative result; VPB ≥ 10/h – excessive ventricular ectopy; nsVT – non-sustained ventricular tachycardia; sVT – sustained ventricular tachycardia.

In our study group the incidence of negative MTWA decreased along with the rise in NYHA scale (NYHA I −60.7%, II −32.1%, III −7.2%). Cardiopulmonary test showed that exercise tolerance measured with peak oxygen consumption (expressed as percentage of predicted values) was worse in patients with abnormal compared to those with negative MTWA: %PeakVO_2_ ml/kg/min (57.4 ± 11.7% vs 64.7 ± 17.0%, p = 0.029), %PeakVO_2_ l/min (58.7 ± 15.4% vs 66.4 ± 17.1%, p = 0.034). The difference was also revealed with regard to chronotropic response which was worse among subjects with abnormal compared to normal MTWA: HRmax (145.5 ± 26.1 bpm vs 160.1 ± 20.9 bpm, p = 0.016), %HRmax (81.4 ± 11.7% vs 88.8 ± 11.0%, p = 0.003). Patients with MTWA(non-) also had higher (reaching statistical significance) concentrations of serum BNP [57.1 pg/ml(min23.3-max343.5) vs 42.2 pg/ml (min6.6-max598.4), p = 0.052] (Table 4).

**Table 4:** Relationship between MTWA result and cardiopulmonary parameters or serum BNP concentration in the analyzed group

	**Analyzed group (n = 92)**	**MTWA(+) (n = 18)**	**MTWA (ind) (n = 18)**	**MTWA(−) (n = 56)**	**MTWA (non -) (n = 36)**	**MTWA(+) vs MTWA(−) p**	**MTWA(non-) vs MTWA(−) p**
**BNP (pg/ml)**	45.1	41.9	64.4	42.2	57.1	0.247	**0.052**
Median	(6.6-598.4)	(23.3-150.8)	(25.1-343.5)	(6.6-598.4)	(23.3-343.5)		
Min-max							
**PeakVO**_**2 **_**(ml/kg/min)**	23.9 ± 6.7	23.1 ± 5.5	21.5 ± 6.9	24.9 ± 6.9	22.3 ± 6.2	0.317	0.072
Mean ± SD							
**%PeakVO**_**2 **_**(ml/kg/min)**	62.3 ± 17.3	57.6 ± 9.6	57.2 ± 13.9	64.7 ± 17.0	57.4 ± 11.7	0.098	**0.029**
Mean ± SD							
**PeakVO**_**2 **_**(l/min)**	1.6 ± 0.6	1.6 ± 0.5	1.4 ± 0.7	1.6 ± 0.6	1.5 ± 0.6	0.845	0.376
Mean ± SD							
**%PeakVO**_**2 **_**(l/min)**	64.0 ± 18.8	57.4 ± 12.7	60.0 ± 18.2	66.4 ± 17.1	58.7 ± 15.4	**0.046**	**0.034**
Mean ± SD							
**HRmax**	154.3 ± 24.0	153.4 ± 27.0	137.1 ± 22.9	160.1 ± 20.9	145.5 ± 26.1	0.280	**0.016**
Mean ± SD							
**%HRmax**	85.9 ± 11.8	85.0 ± 10.8	77.6 ± 11.8	88.8 ± 11.0	81.4 ± 11.7	0.200	**0.003**
Mean ± SD							
**VE/VCO**_**2**_**slope**	32	32.5	42	32	36	0.118	0.061
Median	(22–60)	(26–60)	(26–59)	(22–58)	(26–60)		
Min-max							

MTWA(+) – positive result; MTWA(ind) – result indeterminate due to patient factors; MTWA(−) – negative result; MTWA(non-) – non-negative result; BNP – brain natriuretic peptide; HRmax – maximal heart rate on exercise; PeakVO_2_ – peak oxygen uptake on exercise; VE/VCO_2_ slope – ventilation/carbon dioxide slope.

As shown in univariate logistic regression analysis (Table 5), malignant VA was the most powerful factor to increase probability of abnormal MTWA (OR = 6.4, p = 0.029). This risk was also over twice as high in males (OR = 2.7, p = 0.021), in patients with any form (malignant and/or potentially malignant) of VA (OR = 2.6, p = 0.49) and in NYHA class > I (OR = 2.06, p = 0.033). Cardiopulmonary parameters also played a significant role, but to a lesser extent: HRmax (OR = 0.97, p = 0.006), %HRmax (OR = 0.94, p = 0.005), %PeakVO_2_ (OR = 0.97, p = 0.042), VE/VO_2_slope (OR = 1.05, p = 0.037). A multivariate logistic regression analysis with regard to the presence of abnormal MTWA confirmed a significant predictive value of sVT (OR = 20.7, p = 0.037) and male gender (OR = 15.9, p = 0.001).

**Table 5:** Factors increasing probability of abnormal MTWA in uni- and multivariate linear logistic regression analysis

	**Univariable analysis**		**Multivariable analysis**	
	**OR (95% CI)**	**p**	**OR (95% CI)**	**p**
**sVT**	6.4 (1.22 – 33.57)	**0.029**	20.74 (1.21 – 355.88)	**0.037**
**Male gender**	2.72 (1.15 – 6.46)	**0.021**	15.98 (3.20 – 79.74)	**0.001**
**VPB ≥ 10/h/nsVT/sVT**	2.56 (1.01 – 6.51)	**0.049**	0.96 (0.23 – 4.05)	0.956
**NYHA**	2.06 (1.06 – 4.00)	**0.033**	2.38 (0.81 – 7.00)	0.115
**VE/VCO**_**2 **_**slope**	1.05 (1.00 – 1.10)	**0.037**	1.06 (0.98 – 1.14)	0.154
**HRmax**	0.97 (0.95 – 0.99)	**0.006**	0.99 (0.96 – 1.02)	0.421
**%HRmax**	0.94 (0.91 – 0.98)	**0.005**	-	-
**%PeakVO**_**2 **_**(l/min)**	0.97 (0.94 – 0.99)	**0.042**	1.03 (0.98 – 1.09)	0.202

sVT – sustained ventricular tachycardia; VPB ≥ 10/h – excessive ventricular ectopy; nsVT – non-sustained ventricular tachycardia; VE/VCO_2_slope – ventilation/carbon dioxide slope; HRmax - maximal heart rate on exercise; PeakVO_2_ – peak oxygen uptake on exercise.

## Discussion

Among the analyzed patients with the chosen forms of CHD characterized by pathology within right ventricle or single ventricle physiology, positive as well as indeterminate MTWA test were present in both 19.6%. These values exceeded those observed in the control group: MTWA(+) was present in 2.2% what corresponded with the results achieved in the previously published studies (2.1% and 5.5%) [23,24]. As there was no available analysis of spectral MTWA phenomenon among adults with CHD, the only comparison could be made to pediatric groups – Cheung et al. [17] assessed MTWA in 49 children after tetralogy of Fallot repair observing 14.2% positive and as much as 37% indeterminate (including those caused by technical artifacts) results. Alexander et al. [16] showed the incidence of positive MTWA in 14% and indeterminate – in 28% out of 50 pediatric patients with CHD, which came close to the outcomes of our study.

It has been proven that the prognostic value of positive and indeterminate results is comparable [15,18,25-27], therefore the majority of analyses interpret currently these results as a common group of non-negative, i.e. abnormal MTWA. In our study abnormal tests were observed in 39.2% of patients, which is markedly less than the values described in the SCD high risk groups of patients with advanced left ventricular non-congenital heart failure and reach 67%-73% [25-27]. Exercise capacity as well as age had a distinctive impact on these populations – over half (51.1%) of the analyzed group presented NYHA I functional class (NYHA II – 39,1%, NYHA III – 9,8%) and majority of them were in their forties, whereas patients assessed in the cited publications were in the sixth or seventh decade of life.

Our study showed that incidence of the malignant VA was significantly higher among patients with abnormal compared to negative MTWA. Moreover, the presence of sVT and, to a lesser extent, presence of any form of VA significantly increased the risk of abnormal MTWA phenomenon. However, similar findings were not confirmed by the study of pediatric CHD patients [17]. Ventricular arrhythmia registered on ambulatory ecg monitoring has not yet been found predictive of SCD in this population, this however might be linked to a low number of such tests performed [1,28]. Nevertheless, the prognostic value of Holter ecg refines when interpreted together with other clinical features conducive to malignant arrhythmia [28].

Therefore, our analysis was extended in order to determine which demographical and clinical features of possible prognostic value for SCD are characteristic for patients with abnormal MTWA. It has been shown that majority of subjects with non-negative result were males and male gender turned out to be a factor of a higher probability of this abnormal electrical phenomenon on multivariate regression analysis. As already proven men are more prone to die of SCD than women [6]. Moreover, male gender is also an acknowledged risk factor for life-threatening VA [29,30] as well as total mortality [1] among patients with CHD.

Patients with different MTWA results did not differ with regard to age. SCD incidence in general population increases one hundred-fold after 35th year of life [6], but in the analyzed population heart disease is present since birth and it is rather the complexity of a given defect that determines patient’s later medical history. Previous cardiosurgery did not influence patients’ MTWA results, but the study group included subjects with various anatomical cardiac anomalies that were operated with various methods. Therefore, patients who underwent a cardiosurgery (e.g. with Fontan method) did not necessarily exhibited better clinical outcome than unoperated patients with other anomalies.

Although widening of QRS complex plays a role in SCD risk stratification [9,31], our study demonstrated no significant difference in this parameter with regard to the presence of MTWA phenomenon.

MTWA outcomes in the context of patients’ exercise tolerance are worth focusing on as heart failure has been found to predict malignant VA and SCD in the population of CHD [7,30]. In this specific group not only left ventricular function becomes impaired, but often also morphologically right systemic ventricle, both of the chambers, or the so called single ventricle may become dysfunctional. We observed that the number of patients with negative MTWA systematically decreased between NYHA I and NYHA III groups. Higher NYHA class was also found to increase the probability of abnormal MTWA phenomenon. It has been proven that deterioration of patients’ functional status assessed with this classification has an adverse prognostic value with regard to SCD in this population [32-34], what suggests a potential role of MTWA assessment in the risk stratification algorithms [10]. Despite some methodological objections (lack of objective assessment), NYHA classification remains the most widely used tool to assess heart failure symptoms, including adults with CHD. Although our study group consisted of patients with complex forms of CHD, over half of them (51,1%) was rated as NYHA class 1. Similar results were obtained by other investigators – NYHA class 1 was reported by Piran et al. [35] in 40% of subjects with single or systemic right ventricle (mean age 28,6 ± 7,8 years), whereas Diller et al. [11] classified 49% of patients with different complex forms of CHD (mean age 33 ± 13 years) as being asymptomatic. Common underestimation of patients’ self-reported functional status is mostly caused by lifelong adaptations to their heart disease and not being aware of slowly progressing exercise intolerance. Additionally, relatively young mean age of our study group (34,2 ± 13,6 years) makes NYHA Class 1 truly more possible as the symptoms of heart failure have not emerged yet. In contrast, the study conducted by Bolger et al. [36] among adults with complex CHD aged 33,5 ± 1,5 years revealed NYHA class 1 and 2 in, respectively, 21% and 59% of subjects. An explanation of such difference when compared to our results may be the fact that study group enrolled by us in almost one third consisted of patients with Ebstein syndrome, who are considered as being asymptomatic over decades [7], whereas this defect was not included into the study protocol by Bolger. Therefore, in such a heterogenous and not numerous population generalization and unequivocal conclusions are hardly possible.

The study design included also more reliable method of exercise tolerance evaluation i.e. cardiopulmonary test. The conclusions drawn from the analysis of these parameters were congruent with the findings based on NYHA classification - a significantly worse exercise capacity was observed among patients with abnormal MTWA compared to those with negative test, which manifested as lower peak oxygen uptake and worse chronotropic response in the former group. The mentioned parameters as well as an increased ventilatory respiratory workload (VE/VCO_2_ slope) raised the probability of abnormal MTWA in the analyzed group. Differences in BNP concentrations among groups varying with regard to MTWA result were not shown in our study.

Elevated pulmonary pressure along with decreased tissue oxygenation also contributes to the pathogenesis of heart failure among CHD patients [10], but since these phenomena often coexist, it is merely possible to analyze them separately [12]. In our trial patients with abnormal MTWA did not diverge from the rest in the incidence of pulmonary hypertension, but they were characterized by a markedly lower blood saturation. Experts’ opinion emphasizes that long-term cyanosis causes myocardial fibrosis which subsequently creates an arrhythmogenic substrate [12,34,37].

### Study limitations

Our cohort is considerably smaller than those selected in previous studies on the prognostic value of MTWA, albeit due to the CHD population characteristics, most of performed clinical trials deal with heterogeneity and limited number of patients. As a consequence, the main limitation of our trial is lack of prospective evaluation of MTWA prognostic value with regard to SCD risk among adults with CHD. Taking into account a small number of patients combined with a low expected number of SCD per year, a statistically well-designed study would require to last at least 10 years [8,37]. Therefore, our analysis will be continued using mortality and arrhythmic end points and for the moment conclusions on the role of MTWA in CHD population can only be drawn indirectly, considering general knowledge on arrhythmic SCD and the data obtained in the study. On the basis of the results presented in our study one may also make an attempt to narrow down the group of adults with CHD who should undergo a careful evaluation of indications for ICD therapy, including MTWA test.

## Conclusions

Abnormal spectral MTWA occurs significantly more often in the population of adults with congenital heart disease characterized by pathology within right ventricle or single ventricle physiology compared to healthy subjects.

The probability of its presence increases in patients with malignant ventricular arrhythmia and among subjects with clinical findings possibly related to lethal arrhythmia including advanced heart failure and cyanosis. Male gander is also associated with abnormal MTWA.

MTWA analysis might be of potential role in risk stratification algorithms for sudden cardiac death in this population, although further investigation in this field is required.

## Competing interests

OT received funding from Servier. All other authors declare that they have no competing interests.

## Authors’ contribution

AC, OT, AB conceived on the study idea, design and performed the data acquisition. AC conducted the literature review. AC, OT and LK conducted the statistical analysis and interpretation of data. AC and OT drafted the manuscript. All authors were involved in revising the article for important intellectual content and gave final approval of the version to be published.

## Pre-publication history

The pre-publication history for this paper can be accessed here:

http://www.biomedcentral.com/1471-2261/13/26/prepub

## References

[B1] Verheugt CL Uiterwaal CS van der Velde ET Meijboom FJ Pieper PG van Dijk AP Vliegen HW Grobbee DE Mulder BJ Mortality in adult congenital heart disease Eur Heart J 2010 31 1220 1229 10.1093/eurheartj/ehq032 20207625

[B2] Oechslin EN Harrison DA Connelly MS Webb GD Siu SC Mode of death in adults with congenital heart disease Am J Cardiol 2000 86 1111 1116 10.1016/S0002-9149(00)01169-3 11074209

[B3] Silka MJ Hardy BG Menashe VD Morris CD A population-based prospective evaluation of risk of sudden cardiac death after operation for common congenital heart defects J Am Coll Cardiol 1998 32 245 251 10.1016/S0735-1097(98)00187-9 9669277

[B4] Trojnarska O Grajek S Katarzyński S Kramer L Predictors of mortality in adult patients with congenital heart disease Cardiol J 2009 16 341 347 19653177

[B5] Triedman JK Should patients with congenital heart disease and a systemic ventricular ejection fraction less than 30% undergo prophylactic implantation of an ICD? implantable cardioverter defibrillator implantation guidelines based solely on left ventricular ejection fraction do not apply to adults with congenital heart disease Circ Arrhythm Electrophysiol 2008 1 307 316 10.1161/CIRCEP.108.805903 19808423

[B6] Zipes DP Camm AJ Borggrefe M Buxton AE Chaitman B Fromer M Gregoratos G Klein G Moss AJ Myerburg RJ Priori SG Quinones MA Roden DM Silka MJ Tracy C **ACC/AHA/ESC 2006 guidelines for management of patients with ventricular arrhythmias and the prevention of sudden cardiac death: a report of the ACC/AHA Task Force and the ESC Committee for Practice Guidelines developed in collaboration with the EHRA and HRS** Circulation 2006 114 e385 484 10.1161/CIRCULATIONAHA.106.178233 16935995

[B7] Baumgartner H Bonhoeffer P De Groot NM de Haan F Deanfield JE Galie N Gatzoulis MA Gohlke-Baerwolf C Kaemmerer H Kilner P Meijboom F Mulder BJ Oechclin E Oliver JM Serraf A Szatamari A Thaulow E Vouhe PR Walma E **ESC guidelines for the management of grown-up congenital heart disease (new version 2010). The task force on the management of grown-up congenital heart disease of the European Society of Cardiology** Eur Heart J 2010 31 2915 57 20801927 10.1093/eurheartj/ehq249

[B8] Walsh EP Cecchin F Arrhythmias in adult patients with congenital heart disease Circulation 2007 115 534 545 10.1161/CIRCULATIONAHA.105.592410 17261672

[B9] Goldberger JJ Cain ME Hohnloser SH Kadish AH Knight BP Lauer MS Page RL Passman RS Siscovick D Stevenson WG Zipes DP AHA/ACC/HRS scientific statement on noninvasive risk stratification for identifying patients at risk for sudden cardiac death Circulation 2008 118 1497 1518 10.1161/CIRCULATIONAHA.107.189375 18833586

[B10] Bolger AP Gatzoulis MA Towards defining heart failure in adults with congenital heart disease Int J Cardiol 2004 97 15 23 15590075 10.1016/j.ijcard.2004.08.005

[B11] Diller GP Dimopoulos K Okonko D Li W Babu-Narayan SV Broberg CS Johansson B Bouzas B Mullen MJ Poole-Wilson PA Francis DP Gatzoulis MA Exercise intolerance in adult congenital heart disease: comparative severity, correlates and prognostic implication Circulation 2005 112 828 835 10.1161/CIRCULATIONAHA.104.529800 16061735

[B12] Trojnarska O Gwizdała A Katarzyński S Katarzyńska A Oko-Sarnowska Z Grajek S Kramer L The BNP concentration and exercise capacity assessment with cardiopulmonary stress exercise test in cyanotic patients with congenital heart diseases Int J Cardiol 2010 139 241 247 10.1016/j.ijcard.2008.10.025 19042041

[B13] Cutler MJ Rosenbaum DS Risk stratification for sudden cardiac death: is there a clinical role for T wave altenans? Hear Rhythm 2009 6 S56 S61 10.1016/j.hrthm.2009.05.025 PMC2761079 19631909

[B14] Bloomfield DM Hohnloser SH Cohen RJ Interpretation and classification of microvolt T-wave alternans tests J Cardiovasc Electrophysiol 2002 13 502 512 10.1046/j.1540-8167.2002.00502.x 12030535

[B15] Van der Avoort CJ Filion KB Dendukuri N Brophy JM Microvolt T-wave alternans as a predictor of mortality and severe arrhythmias in patients with left-ventricular dysfunction: a systemic review and meta-analysis BMC Cardiovascular Disord 2009 9 5 10.1186/1471-2261-9-5 PMC2653469 19175926

[B16] Alexander ME Cecchin F Huang KP Berul CI Microvolt T-wave alternans with exercise in pediatrics and congenital heart disease: limitations and predictive value Pacing Clin Electrophysiol 2006 29 733 741 10.1111/j.1540-8159.2006.00427.x 16884509

[B17] Cheung MM Weintraub RG Cohen RJ Karl TR Wilkinson JL Davis AM T wave alternans threshold late after tetralogy of fallot J Cardiovasc Electrophysiol 2002 13 657 661 10.1046/j.1540-8167.2002.00657.x 12139287

[B18] Kaufman ES Bloomfield DM Steinman RC Namerow PB Costantini O Cohen RJ Bigger JT Jr “Indeterminate” microvolt T wave alternans tests predict high risk of death or sustained ventricular arrhythmias in patients with left ventricular dysfunction J Am Coll Cardiol 2006 48 1399 1404 10.1016/j.jacc.2006.06.044 17010802

[B19] Diller GP Dimopoulos K Broberg CS Kaya MG Naghotra US Uebing A Harries C Goktekin O Gibbs JS Gatzoulis MA Presentation, survival prospects, and predictors of death in eisenmenger syndrome: a combined retrospective and case–control study Eur Heart J 2006 27 1737 1742 10.1093/eurheartj/ehl116 16793921

[B20] Guidelines for the diagnosis and treatment of pulmonary hypertension The task force for the diagnosis and treatment of pulmonary hypertension of the ESC and ERS, endorsed by the ISHLT Eur Heart J 2009 30 2493 2537 19713419 10.1093/eurheartj/ehp297

[B21] Wasserman K Hansen JE Sue DY Principles of exercise testing and interpretation 1986 Philadelphia, PA: Lea and Febiger

[B22] Bigger JT Jr Fleiss JL Kleiger R Miller JP Rolnitzky LM The relationships among ventricular arrhythmias, left ventricular dysfunction, and mortality in the 2 years after myocardial infarction Circulation 1984 69 250 258 10.1161/01.CIR.69.2.250 6690098

[B23] Narayan SM Bayer JD Lalani G Troyanova NA Action potential dynamics explain vulnerability in human heart failure: a clinical study implicating abnormal calcium handling J Am Coll Cardiol 2008 52 1782 1792 10.1016/j.jacc.2008.08.037 19022157 PMC2613952

[B24] Weber S Thillmanns H Waldecker B Prevalence of TWA in healthy subjects Pacing Clin Electrophysiol 2003 26 49 52 10.1046/j.1460-9592.2003.00149.x 12685139

[B25] Costantini O Hohnnloser SH Kirk MM Lerman BB Baker JH 2nd Sethuraman B Dettmer MM Rosenbaum DS The ABCD (alternans before cardioverter defibrillator) trial: strategies using T-wave alternans to improve efficiency of sudden cardiac death prevention J Am Coll Cardiol 2009 53 471 479 10.1016/j.jacc.2008.08.077 19195603

[B26] Bloomfield DM Steinman RC Namerow PB Parides MK Davidenko J Kaufman ES Shinn T Curtis A Fontaine J Holmes D Russo A Tang C Bigger JT Jr Microvolt T-wave alternans distinguishes between patients likely and patients not likely to benefit from implanted cardiac defibrillator therapy Circulation 2004 110 1885 1889 10.1161/01.CIR.0000143160.14610.53 15451804

[B27] Gehi AK Stein RH Metz LD Gomez A Microvolt T-wave alternans for the risk stratification of ventricular tachyarrhythmic events. A meta-analysis J Am Coll Cardiol 2005 46 75 82 10.1016/j.jacc.2005.03.059 15992639

[B28] Khairy P Aboulhosn J Gurvitz MZ Opotowsky AR Mongeon FP Kay J Valente AM Earing MG Lui G Gersony DR Cook S Ting JG Nickolaus MJ Webb G Landzberg MJ Broberg CS Arrhythmia burden in adults with surgically repaired tetralogy of fallot A multi-institutional study. Circulation 2010 122 868 875 10.1161/CIRCULATIONAHA.109.928481 20713900

[B29] Khairy P Harris L Landzberg MJ Fernandes SM Barlow A Mercier LA Viswanathan S Chetaille P Gordon E Dore A Cecchin F Sudden death and defibrillators in transposition of the great arteries with intra-atrial baffles. A multicenter study Circ Arrhythmia Electrophysiol 2008 1 250 257 10.1161/CIRCEP.108.776120 19808416

[B30] Tsai SF Chan DP Ro PS Boettner B Daniels CJ Rate of inducible ventricular arrhythmia in adults with congenital heart disease Am J Cardiol 2010 106 730 736 10.1016/j.amjcard.2010.04.036 20723654

[B31] Schwerzmann M Salehian O Harris L Siu SC Williams WG Webb GD Colman JM Redington A Silversides CK Ventricular arrhythmias and sudden death in adults after a mustard operation for transposition of the great arteries Eur Heart J 2009 30 1873 1879 10.1093/eurheartj/ehp179 19465439

[B32] Khairy P Fernandes SM Mayer JE Jr Triedman JK Walsh EP Lock JE Landzberg MJ Long-term survival, modes of death, and predictors of mortality in patients with fontan surgery Circulation 2008 117 85 92 10.1161/CIRCULATIONAHA.107.738559 18071068

[B33] Diller GP Giardini A Dimopoulos K Garqiulo G Muller J Derrick G Giannakoulas G Khambadkone S Lammers AE Picchio FM Gatzoulis MA Hager A Predictors of morbidity and mortality in contemporary fontan patients: results from a multicenter study including cardiopulmonary exercise testing in 321 pts Eur Heart J 2010 31 3073 3083 10.1093/eurheartj/ehq356 20929979

[B34] Perloff JK, Child JS Congenital heart disease in adults 1998 2 Philadelphia: WB Saunders Co

[B35] Piran S Veldtman G Siu S Webb GD Liu PP Heart failure and ventricular dysfunction in patients with single or systemic right ventricles Circulation 2002 105 1189 1194 10.1161/hc1002.105182 11889012

[B36] Bolger AP Sharma R Li W Leenarts M Kalra PR Kemp M Coats AJ Anker SD Gatzoulis MA Neurohormonal activation and the chronic heart failure syndrome in adults with congenital heart disease Circulation 2002 106 92 99 10.1161/01.CIR.0000020009.30736.3F 12093776

[B37] Silka MJ Bar-Cohen Y Should patients with congenital heart disease and a systemic ventricular ejection fraction less than 30% undergo prophylactic implantation of an ICD? patients with congenital heart disease and a systemic ventricular ejection fraction less than 30% should undergo prophylactic implantation of an implantable cardioverter defibrillator Circ Arrhythm Electrophysiol 2008 1 298 306 10.1161/CIRCEP.108.801522 19808422

